# Editorial: Lifestyle and healthy aging to prevent cognitive decline and dementia

**DOI:** 10.3389/frdem.2025.1694494

**Published:** 2025-10-07

**Authors:** Kristin R. Krueger, Mathieu Hainselin, Ayse Kuspinar, Breno José Alencar Pires Barbosa, Celeste A. de Jager Loots

**Affiliations:** ^1^Rush University Medical Center, Chicago, IL, United States; ^2^CRPCPO, UR UPJV 7273, Université de Picardie Jules Verne, Amiens, France; ^3^School of Rehabilitation Science, McMaster University, Hamilton, ON, Canada; ^4^Medical Sciences Center, Federal University of Pernambuco, Recife, Brazil; ^5^Neurology Division, Hospital das Clínicas, Federal University of Pernambuco, Empresa Brasileira de Serviços Hospitalares, Recife, Brazil; ^6^Ageing Epidemiology Research Unit, School of Public Health, Imperial College London, London, United Kingdom; ^7^FINGERS Brain Health Institute, Solna, Sweden

**Keywords:** healthy aging, cognitive activity, older adults, dementia risk reduction, physical activity, social activity

## Introduction

We now have evidence that lifestyle behaviors can reduce the risk of cognitive decline and dementia even among older adults with high genetic risk (Lourida et al., 2019). With this in mind, we created this Research Topic to expand our knowledge of targets for lifestyle interventions to reduce dementia risk. Our aim was to cast a net wide enough to attract submissions from around the world both from established and from newer researchers. We wanted to highlight smaller studies that approach this topic from novel perspectives that in turn may inspire new ideas that might expand our conceptualizations for dementia prevention. Finally, we were interested in how multiple factors or the interaction of factors may influence cognitive function, cognitive decline, and dementia.

The resulting collection in this Research Topic is made up of 30 articles, with authors hailing from 6 continents and 19 countries. The contents of manuscripts covered a wide range of topics that can be classified broadly into the following categories: Physical Activity, Cognitive Activity/Training, Depression/Social Support, Diet/Alcohol, Obesity/Waist Circumference/Diabetes, Enriched Environments, and Sex/Gender/HRT.

## Physical activity

Physical activity is one of the most researched lifestyle factors for healthy aging in the last two decades (Moreno-Agostino et al., 2020). With a growing aging population, examining how physical activity affects cognitive health is more important than ever. In this Research Topic, we were pleased to include an interesting range of articles on physical activity. A network meta-analysis (Han et al.) identified that resistance training and mind-body exercises, such as Tai Chi, were effective in improving global cognitive function, executive function and memory in healthy older adults. Similarly, a 12-week Tai Chi intervention (Lin L. et al.) demonstrated efficacy in improving overall cognitive function, executive function, and memory in older adults with mild cognitive impairment. Furthermore, a mouse model of Alzheimer's disease (Perez Garcia et al.) showed that physical exercise and BCI-838 drug administration, improved recognition memory and enhanced adult hippocampal neurogenesis. Despite the many benefits of physical activity, a Mendelian randomization study (Li Y. et al.) suggested that the dose of physical activity is also important to consider in relation to cognitive performance. The relationship between physical activity dose and cognitive performance may be non-linear, with excessive amounts of moderate-to-vigorous physical activity resulting in unfavorable cognitive outcomes. Last, a study using structural equation modeling and fuzzy set qualitative comparative analysis the authors (Li W. et al.) reported that physical activity behavior is affected by multiple factors, including social support, motivation, and physical literacy. In summary, although physical activity plays an important role in cognitive health, the relationship is a complex one. Intensity, duration and mode of physical activity, as well as the individual's social and psychological situation, are important factors to consider.

## Cognitive activity/cognitive training

Although cognitive activity is a cornerstone of dementia prevention, the field continues to grapple with the challenges of intervention efficacy, adherence, and ecological validity. Articles in this special topic contribute to this ongoing discussion by examining how, for whom, and under what conditions cognitive training may be optimized.

To address the critical issue of participant engagement in long-term trials, a Cognitive Training Support Programme (CTSP) has been developed to supplement computerized cognitive training (CCT) interventions (de Jager Loots et al.). Moving beyond the training tasks themselves, the CTSP leverages a behavior change framework (Capability, Opportunity, Motivation) to improve adherence through group sessions that educate, support, and motivate participants. This global approach recognizes that an intervention's success depends as much on its supportive framework as its content.

In a novel approach to making cognitive activity more engaging, improvisational theater has been established as a feasible and meaningful cognitive activity (Krueger et al.). Neuropsychology experts and trainees rated improv exercises as effectively engaging multiple cognitive abilities, particularly sustained attention and processing speed. This work opens promising avenues for developing interventions that are not only cognitively stimulating but also inherently social and enjoyable, potentially boosting adherence and wellbeing.

Finally, a new meta-analysis examines the synergy of combining Cognitive Training (CT) with transcranial Direct Current Stimulation (tDCS; Lv et al.). The authors showed this combination significantly improves working memory in healthy older adults compared to control conditions. Crucially, a dose-dependent effect was identified, with protocols using a 2 mA intensity and lasting 10 or more sessions yielding the best results. This research underscores the potential of combining behavioral and neuromodulation techniques while highlighting the critical need for rigorous parameter optimization to maximize therapeutic benefits.

## Depression and social support

Depression is reported to be both a predictor and a consequence of cognitive decline but may also be a confounder in diagnosis of mild cognitive impairment (MCI) and Alzheimer's disease (AD). There are three articles related to depression in this collection, two from China where depression in older adults over 45 years of age is reported to be particularly prevalent.

Firstly, one study showed that a grip strength measure is associated with depressive symptoms and may be mediated by cognitive function, especially in men (Wang, Wu et al.). The authors suggest that targeted interventions, possibly focusing on enhancing grip strength and cognitive function, should be explored as they may help in reducing the risk and severity of depressive symptoms in middle-aged and older adults.

Secondly, a study in rural older adults (14% of whom had depression) looked at effects of perceived social support on cognitive function (Gui et al.). The strongest sources of perceived support (defined as providing emotional support or satisfaction, feeling respected, supported and understood within social networks) were ranked 1) children, 2) neighbors 3) a spouse, but analysis of cognitive function found that support from friends ranked third after children and neighbors. A combination of two or more sources of support was associated with improved cognition, especially for males and those financially well-off. Older age and depression were related to lower cognitive function. Social support is seen to act as a stress buffer and provide cognitive reserve.

Finally, a study from Korea (Lee et al.) supporting the hypothesis of depression as a risk factor for cognitive decline, looked at items from the Geriatric Depression Scale (GDS) in combination with the total consortium to establish a registry for Alzheimer's disease (CERAD) score and with the MMSE. Analysis revealed that the GDS ‘helplessness' item was a significant predictor for MCI diagnosis and was related to decreased cognitive function on the CERAD total score, but only in those individuals reporting lower levels of physical activity. MMSE plus ‘helplessness' improved diagnostic accuracy for MCI as opposed to MMSE score alone.

## Diet/alcohol

Dietary and alcohol intake considerations are now main targets in the quest for living longer with preserved cognitive functioning. This selection of articles includes several novel approaches to increase our understanding of dietary intake in diverse settings. A 10-year cohort study conducted in China (Wang, Qiao et al.) demonstrated that more years of education were associated with the diet of older adults, but only in the slow cognitive decline group. Food diversity partially mediates the relationship between years of education and cognitive trajectories. Perhaps more noteworthy, food variety had a mediating effect on the relationship between years of education and changes in cognitive function. The authors describe how a higher level of education may influence essential habits (e.g., how we choose and prepare food) that in turn affects our biology and cognition.

Findings on alcohol intake and its relationship to health has had many ups and downs over the last two decades, with the most current statement by the World Health Organization indicating that “Alcohol consumption, even at low levels can bring health risks, but most alcohol related harms come from heavy episodic or heavy continuous alcohol consumption.” (World Health Organization, 2024). A population-based study from NHANES 2011–2014 (Chen et al.) demonstrated that although the relationship is complex, there was an overall negative correlation between alcohol intake and cognitive impairment in older adults. However, this relationship was non-linear and indicative that primarily heavy drinking was associated lower cognitive functioning over time.

Despite the increase in the scientific study of dietary intake and its relationship to cognition, messages to the general public may still be lacking. A cross-sectional study of informal caregivers' knowledge and motivation to adhere to the MIND diet (Guzman and Aguiñaga) demonstrated that knowledge and adherence was generally low. The authors describe the barriers to consuming the MIND diet in this group and offer possible solutions to intervene.

Finally, an animal intervention of two plant-based supplements that have anti-inflammatory properties (Boualam et al.) provided some insight into dietary effects on aging well. After 2 years, both *Mentha rotundifolia* (L.) Huds. and *Salvia officinalis* L. hydrosols mitigated aging related comorbidities in rats. That is, the rats who consumed the plant-based supplements had better physical and behavioral functioning compared to the rats who were not treated.

## Obesity/waist/diabetes

Not only is Diabetes a significant risk factor for dementia (Livingston et al., 2024) worldwide, but there's growing evidence that insulin resistance and allostatic load could play a role in AD's pathology and risk stratification in MCI (De Felice et al., 2022; Barbosa et al., 2024). Dysregulated glycemic pathways and chronic hypercortisolism could elevate overall cardiovascular risk, leading to stroke and small vessel disease, two major causes of vascular cognitive impairment (Hou et al., 2019); Moreover, elevated glycated hemoglobin (HbA1c) is a common finding in individuals with metabolic syndrome, who usually have hypertension, dyslipidemia, central obesity, and/or altered waist/hip ratios. Metabolic syndrome is associated with cognitive dysfunction (Akbaraly et al., 2010; Segura et al., 2009), and some high-quality articles included in the present Research Topic could highlight some pathways toward mechanisms and preventive strategies.

A longitudinal study from Portugal (Canário et al.) examined whether higher levels of glycated hemoglobin affect long-term brain structure in key memory regions. Gray matter volume was measured at two times—initially and after about 7 years. Findings revealed that higher baseline HbA1C levels were linked to decreases in gray matter volume across all three main memory areas, even in the absence of mild cognitive impairment signs. Interestingly, these brain changes remained even when patients greatly improved their glycemic control. This suggests that high HbA1C levels early on might be permanently associated with long-term atrophy in the medial temporal cortex, highlighting the need for early, aggressive treatment.

Expanding on studies of HbA1c and cognitive impairment, research from South Korea (Sook Kim et al.) investigated the link between HbA1c levels and cognitive decline in older Koreans without dementia. The authors used data from a community-based Ansan cohort (2009–2010) consisting of 853 participants aged 59 and above. Cognitive function was measured using MMSE and MoCA, then categorized as normal or impaired. Multiple logistic regression analyzed the relationship between HbA1c and impairment, adjusting for various factors. In women, higher HbA1c was a risk factor for lower MMSE scores; compared to HbA1c ≤ 5.6%, adjusted odds ratios for levels of 5.7–6.4% and ≥6.5% were 2.16 and 2.96, respectively. The authors concluded that better glycemic control could help lower the risk of cognitive impairment in older adults, highlighting the need for further research into sex differences.

A Chinese report (Wu, Zhu et al.) with 3,807 participants aged 45 and older from 2010 to 2020 used TICS-10 to assess cognition. The group-based trajectory model (GBTM) explored cognitive change heterogeneity. Abdominal obesity was measured by baseline waist circumference (WC), analyzed via multinomial logistic regression for its association with cognitive decline. Controversially, in this study, abdominal obesity had a significant protective effect on cognitive decline in Chinese middle-aged and older adults, with HDL-C playing a mediating role in the relationship between abdominal obesity and cognitive decline.

In the same context, another Chinese group (Lin J. et al.) evaluated a more advanced metric: the Weight-adjusted Waist Index (WWI), which incorporates changes in body composition, such as muscle and fat mass, to offer a more thorough assessment of centripetal obesity. The study involved 1,392 older Chinese men aged 65 and above with complete data available. After considering all possible confounding variables, the analysis found a statistically significant positive association between WWI and cognitive decline.

## Hypertension and blood flow

Hypertension is probably the single most important modifiable risk factor for cardiovascular and cerebrovascular disease, thus contributing to a broad spectrum of cognitive vascular impairment ranging from preclinical “at-risk” individuals to MCI and vascular dementia (Ter Telgte et al., 2018; Barbosa et al., 2022; van der Flier et al., 2018). The present Research Topic included three articles that brought a significant contribution to the field by examining diverse populations.

One study (Horan et al.) analyzed data from the Irish Longitudinal Study on Aging (TILDA). The authors examined the independent and moderating effects of increased arterial stiffness (AS) and decreased cerebral blood flow (CBF) on hippocampal volume (HV) in a large MRI sample of community-dwelling older adults (*N* = 395). Increased arterial stiffness and reduced CBF were not independently linked to smaller HV. However, when combined, persistently elevated AS and reduced CBF are associated with smaller HV. These findings imply a delayed effect in the relationship between arterial stiffness and hippocampal volume.

A group from India (Parandiyal et al.) included 95 hypertensive participants aged 60 years and older from cardiology and medicine outpatient services at a tertiary care hospital to assess the characteristics of hypertension and its impact on cognitive functions. The features of hypertension and cognitive functions were evaluated using a semi-structured proforma and the Adenbrooke's Cognitive Examination Hindi version, respectively. Although there was a significant difference in cognitive functions related to the duration and status of hypertension, a notable difference was observed in the attention and fluency domains of cognitive function based on hypertension status. Additionally, there was a general decline in cognitive domains associated with both hypertension status and the duration of hypertension.

Finally, Chinese investigators (Wu, Yang et al.) reported on the measurement of inter-arm blood pressure difference (IABPD) and cognitive impairment using data from the Cohort Study of the Health Status of Guizhou Rural Older Adults in China. A multi-stage cluster sampling method was used to select 1,088 rural elderly individuals aged 60 years and older. The cognitive function of participants was assessed with the Mini-Mental State Examination (MMSE). Bilateral blood pressure was measured simultaneously with an automated device, and the IABPD was calculated. Multivariable regression analysis showed that an inter-arm systolic blood pressure difference (IASBPD) of 10 mmHg or more was independently linked to lower MMSE scores and an increased risk of cognitive impairment.

## Enriched environments, individual differences

There are several activities or contexts that provide enriching environments that are associated with better cognitive functioning in older adults. The field has gained traction with the concept of cognitive reserve, the presumed ability of the brain to continue to function well despite accumulated neuropathology (Wilson et al., 2019). Nonetheless, the extent to which enriched environments can designed and wielded to affect the course of cognition over time is open for more improvement. This edition reviews an interesting variety of enriched environments that are both timely and productive. One study demonstrates to what extent cognitive processing speed can be improved after cochlear implantation (Mosnier et al.). Perhaps one of the most novel articles in this category reports on the potential cognitive benefits of older adults engaging in foreign language learning training (Schultz et al.). Environmental enrichment was also associated with memory-related functional brain activity patterns in older adults (Hass et al.). A very thoughtful study examined the impact of social activity on the relationship between functional mobility and cognition (Wang, Chen et al.). Finally, there was evidence that cognitive reserve may produce neurophysiological markers as measured by EEG (Katayama et al.).

## Sex/gender/HRT

Individual differences that affect cognition, such as sex and its intersection with aging, hormones and lifestyle variables, have been included in this series. One study reported on the associations of hormone replacement therapy, menopausal age and lifestyle variables (Watermeyer et al.). In a provocative study with mice, researchers explored the impact of exercise and chronic stress on males and females across the lifespan (Sullens et al.).
